# Assessing Health Literacy Among East Asian Older Populations

**DOI:** 10.1093/geroni/igaa057.2955

**Published:** 2020-12-16

**Authors:** Taka Yamashita

**Affiliations:** University of Maryland, Baltimore, Maryland, United States

## Abstract

Health literacy assessment tools that are designed and validated specifically for East Asian older adults are limited. This session reviews the existing health literacy assessment tools and discusses possible applications in East Asian older adults. As an alternative to the population-level health literacy assessment, the general literacy measures and assessment approach in the Program for International Assessment of Adult Competencies (PIAAC) data are discussed. As a case demonstration, the PIAAC literacy measures in Japan and Korea are summarized and evaluated for the applicability in aging and health research with East Asian older adults.

